# The relationship between dietary flavonoid intake and hypertension: a cross-sectional study from NHANES

**DOI:** 10.3389/fcvm.2025.1518549

**Published:** 2025-05-30

**Authors:** Yueyue Niu, Xingjuan Chen, Lu Xiao, Weina Li, Ling Feng, Aikeremu Aierken

**Affiliations:** ^1^ Cadres Health Protection Department, China Academy of Chinese Medical Sciences Guang'anmen Hospital, Beijing, China; ^2^ Chinese and Western Medicine Collaborative Diagnosis and Treatment Medical Center, Xinjiang Uygur Autonomous Region People’s Hospital, Urumqi, Xinjiang, China

**Keywords:** flavonoids, hypertension, NHANES, cross-sectional study, dietary intake

## Abstract

**Objective:**

To evaluate the association between dietary flavonoid intake and hypertension using nationally representative data, considering increasing global hypertension prevalence and inconsistent evidence on flavonoids’ protective effects.

**Methods:**

This cross-sectional study analyzed 8,054 adults aged ≥20 years from NHANES 2007–2010 and 2017–2018. Flavonoid intake was assessed through two 24-hour dietary recalls and categorized into quartiles. Hypertension was defined by blood pressure ≥130/80 mmHg or self-reported diagnosis/medication use. Logistic regression models with progressive adjustment, restricted cubic spline regression for dose-response relationships, and subgroup analyses were conducted, accounting for complex sampling design.

**Results:**

After full adjustment, participants in the highest quartile of total flavonoid intake showed 25% lower odds of hypertension compared to the lowest quartile (OR = 0.75, 95% CI: 0.60–0.93, *p* = 0.01). Among flavonoid subclasses, anthocyanidins (OR = 0.74, 95% CI: 0.58–0.93) and flavan-3-ols (OR = 0.76, 95% CI: 0.62–0.93) demonstrated the strongest protective associations. Significant effect modifications were observed for age (*p* for interaction = 0.01), hyperlipidemia (*p* for interaction <0.0001), and cardiovascular disease status (*p* for interaction =0.01), with stronger protective effects in younger adults and those without metabolic disorders.

**Conclusion:**

Moderate dietary flavonoid intake, particularly anthocyanidins and flavan-3-ols, is inversely associated with hypertension risk. These associations vary significantly by age and metabolic status, suggesting potential for individualized dietary recommendations for hypertension prevention.

## 1 Introduction

Hypertension remains one of the most prevalent chronic diseases globally and a leading risk factor for cardiovascular and cerebrovascular diseases. According to the World Health Organization's 2023 Global report on hypertension, over one billion people worldwide currently live with hypertension, making it a significant public health concern globally (1).

Flavonoids are a diverse class of polyphenolic compounds abundantly found in plant-based foods, known for their potent antioxidant and anti-inflammatory properties (2, 3). Flavonoids are classified into six major subclasses: flavonols, flavones, isoflavones, flavanols, anthocyanins, and flavanones. These compounds are richly present in fruits, vegetables, tea, cocoa, and red wine (4, 5). Flavonoids exert their biological activities through multiple mechanisms, including free radical scavenging, modulation of oxidative stress-related enzymes, inhibition of pro-inflammatory cytokine production, and regulation of endothelial function (6).

The relationship between flavonoids and hypertension remains inconsistent across studies. Several experimental investigations demonstrate the potential antihypertensive effects of flavonoids. Research demonstrates that flavonoids can lower blood pressure by enhancing nitric oxide (NO) bioavailability, reducing angiotensin-converting enzyme (ACE) activity, and improving vascular endothelial function (7–9). However, Zhu in their PRISMA-compliant meta-analysis of six randomized clinical trials involving 472 participants, failed to establish a significant association between anthocyanin supplementation and blood pressure reduction, finding no meaningful effect on either systolic blood pressure (10).

Given the inconsistency in existing research, this study aims to evaluate the association between flavonoid intake and hypertension using nationally representative data from the National Health and Nutrition Examination Survey (NHANES) 2007–2010 and 2017–2018, employing a cross-sectional study design. This research will contribute valuable evidence to inform dietary strategies for hypertension prevention and potentially provide new perspectives for clinical nutritional interventions.

## 2 Methods

### 2.1 Study population

**Figure 1:**
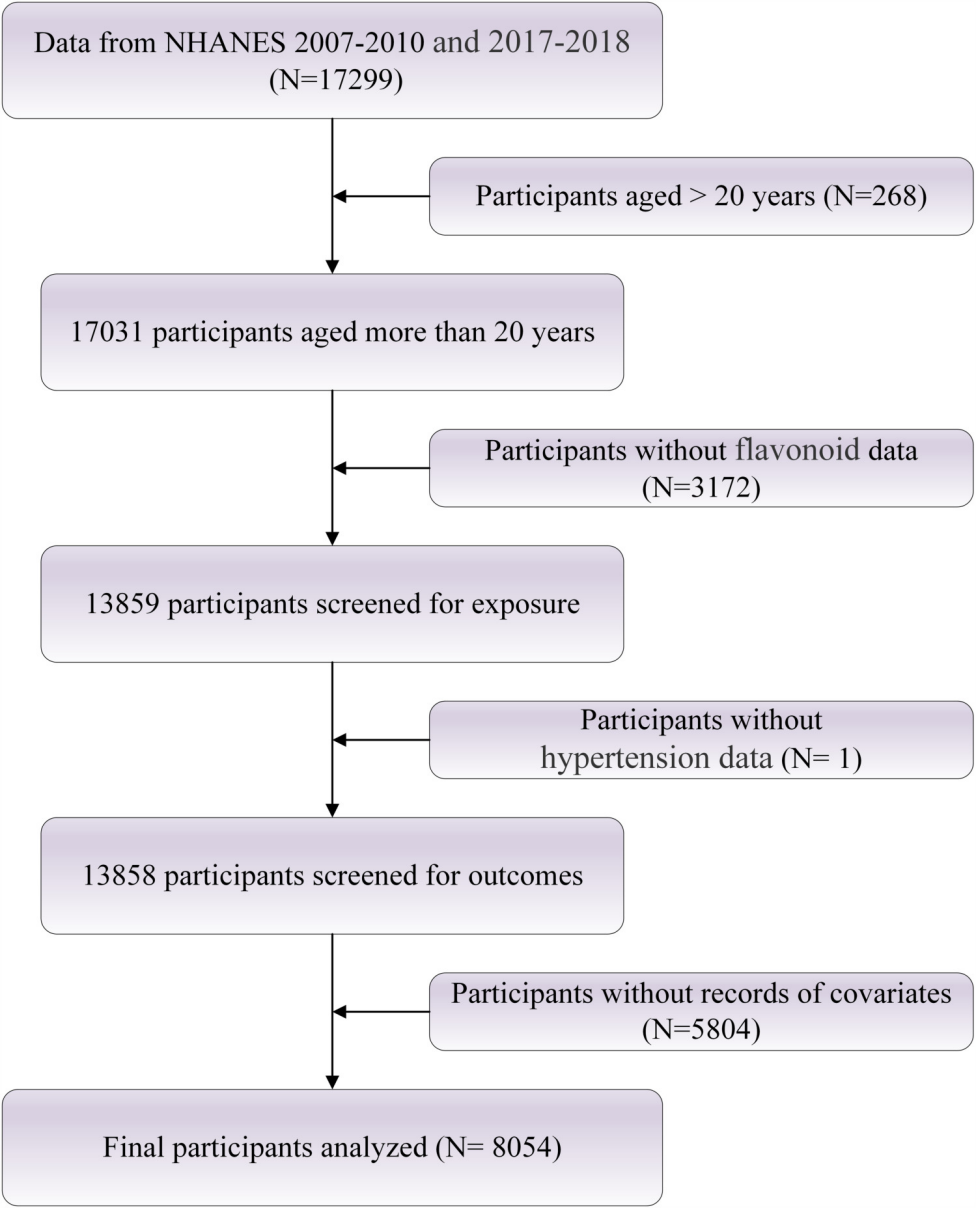
Flowchart of study population selection.

The NHANES, conducted by the Centers for Disease Control and Prevention (CDC), is a critical and ongoing study designed to systematically assess the health and nutritional status of the U.S. population. NHANES combines self-reported questionnaires, comprehensive physical examinations, and laboratory tests to collect multidimensional health data, including dietary intake, prevalence of chronic diseases, and various biological markers. All participants provided written informed consent prior to enrollment, ensuring that they fully understood and agreed to the study's objectives and procedures. The study protocol was approved by the Ethics Review Board of the National Center for Health Statistics, and further details are available at https://www.cdc.gov/nchs/nhanes/index.html. Participants for the present analysis were drawn from the 2007–2010 and 2017–2018 NHANES cycles and included adults aged 20 years and older. To enhance the validity and reliability of the sample, individuals younger than 20 years and those with incomplete data were excluded. A total of 8,054 participants were ultimately included in the study. The detailed selection process is illustrated in Figure 1.

### 2.2 Dietary flavonoid intake assessment

In the NHANES study, dietary intake data were collected through two 24-hour dietary recall interviews. The first recall was conducted in person at the Mobile Examination Center (MEC), followed by a second recall via telephone approximately three to ten days later. All interviewers underwent a rigorous one-week training program, including supervised practice sessions, to ensure they could independently perform field interviews with accuracy and consistency. To standardize nutrient intake estimations, the Food and Nutrition Database for Dietary Studies (FNDDS), maintained by the U.S. Department of Agriculture (USDA), was used (11). Flavonoid intake information was derived from FNDDS versions 4.1 and 5.0, which include data on 29 flavonoids categorized into six primary subclasses: isoflavones, anthocyanidins, flavan-3-ols, flavanones, flavones, and flavonols. Specifically, isoflavones consist of daidzein, genistein, and glycitein; anthocyanidins encompass cyanidin, delphinidin, malvidin, pelargonidin, peonidin, and petunidin; flavan-3-ols include compounds such as epicatechin, catechin, and theaflavins; flavanones comprise eriodictyol, hesperetin, and naringenin; flavones include apigenin and luteolin; and flavonols are represented by isorhamnetin, kaempferol, myricetin, and quercetin. The overall flavonoid intake was calculated by summing the amounts across all six subclasses (12). Daily flavonoid consumption (expressed as milligrams per 100 grams of food or beverage per day) was determined by averaging the results from the two dietary recall interviews, thereby minimizing potential inconsistencies related to recall frequency.

### 2.3 Assessment of hypertension

Hypertension was identified based on both questionnaire responses and physical examination findings if participants satisfied any of the following criteria: (1) an average systolic blood pressure (SBP) of at least 130 mmHg or an average diastolic blood pressure (DBP) of at least 80 mmHg; (2) a self-reported affirmative response to the question “Have you ever been advised to take prescribed medication for high blood pressure?”; or (3) a self-reported affirmative answer to the question “Have you ever been diagnosed with hypertension?”. All blood pressure measurements were conducted at the MEC. The average blood pressure was determined following specific procedures: diastolic readings recorded as zero were excluded from the calculation; if all diastolic measurements were zero, the average was recorded as zero. In cases where only a single blood pressure measurement was available, that value was considered the average. When multiple readings were available, the first measurement was systematically omitted from the average calculation.

### 2.4 Covariates

Based on previous studies, we identified several potential confounders associated with dietary flavonoid intake and nocturia. These included sociodemographic factors (sex, age, race [Mexican American, other Hispanic, non-Hispanic White, non-Hispanic Black, and other races], marital status [married/living with partner, never married, divorced/separated/widowed], educational level [<high school, high school graduate or equivalent, ≥some college], and poverty income ratio [PIR, low: ≤1.3; middle: 1.3–3.5; high: ≥3.5]), lifestyle behaviors (smoking status, drinking status, and physical activity), anthropometric measures (BMI, categorized as <18.5, 18.5–24.9, 25.0–29.9, and ≥30 kg/m²), dietary factors (DASH score and total intake of energy, protein, carbohydrates, sugar, and fat) (13), and clinical conditions [diabetes, hyperlipidemia, and cardiovascular disease (CVD)].

All covariates were collected through structured computer-assisted personal interviews. Smoking status was categorized as never (<100 cigarettes lifetime), former (≥100 cigarettes but not currently smoking), or current (≥100 cigarettes and still smoking). Drinking status was defined as: never (<12 drinks lifetime), former (≥12 drinks in lifetime but not in past year), mild (≤1 drink/day for females, ≤2 for males), moderate (≤2 drinks/day for females, ≤3 for males or binge drinking 2–4 times/month), and heavy (≥3 drinks/day for females, ≥4 for males or binge drinking ≥5 times/month).

Diabetes was defined by any of the following: self-reported physician diagnosis, HbA1c ≥ 6.5%, fasting plasma glucose ≥7.0 mmol/L, random glucose ≥11.1 mmol/L, or 2-hour OGTT glucose ≥11.1 mmol/L. CVD was defined as a self-reported history of at least one of the following: congestive heart failure, coronary heart disease, angina pectoris, or myocardial infarction.

Dietary intake was assessed using one or two 24-hour dietary recalls conducted by trained interviewers. The first recall was in person, and the second (3–10 days later) was via telephone. Food and nutrient intakes were estimated using the USDA's Automated Multiple-Pass Method (AMPM) and calculated based on the FNDDS (14, 15). For participants completing two recalls, a validated multiple-day averaging method was used to estimate usual nutrient intake.

### 2.5 Statistical analysis

The baseline characteristics of the participants were stratified by hypertension status. Continuous variables were presented as means and standard errors, while categorical and ordinal variables were expressed as frequencies and percentages. All analyses accounted for the complex sampling design and national representativeness of the NHANES through the application of appropriate survey weights. Logistic regression models were used to examine the association between flavonoid intake and the prevalence of hypertension. Flavonoid intake was categorized into quartiles (Q1–Q4). Three models were constructed: the crude model was unadjusted; model 1 was adjusted for age, sex, and race; and model 2 was further adjusted for marital status, PIR, education level, drinking status, smoking status, PA, BIM, DASH score, energy intake, protein intake, carbohydrate intake, sugar intake, fat intake, and histories of diabetes, hyperlipidemia, and cardiovascular disease. The dose–response relationship between flavonoid intake and hypertension was explored using restricted cubic spline (RCS) regression based on multivariable logistic models, with nonlinearity assessed by *p*-values. Subgroup analyses were also performed to evaluate potential effect modifications. A two-sided *P*-value < 0.05 was considered indicative of statistical significance. All statistical analyses were conducted using R software (version 4.2.3).

## 3 Results

### 3.1 Baseline characteristics of the study population

The baseline characteristics of participants with and without hypertension are presented in Table 1. A total of 8,054 individuals were included, comprising 2,897 with hypertension and 5,157 without hypertension. Compared with normotensive individuals, participants with hypertension were older(*p* < 0.0001), more likely to be male (*p* = 0.01), and had a different racial distribution, with a higher proportion of Non-Hispanic Blacks and fewer Mexican Americans (*p* < 0.0001). They exhibited less favorable lifestyle behaviors, including lower rates of heavy and moderate drinking, higher proportions of mild drinking and former drinking, more former smokers, and higher prevalence of insufficient physical activity (all *p* < 0.0001). Obesity was notably more common among hypertensive participants (*p* < 0.0001). They consumed less total energy, carbohydrates, and sugars (all *p* ≤ 0.01), but had slightly higher total flavonoid and flavan-3-ols intake (both *p* = 0.05) and lower isoflavone intake (*p* = 0.03). The prevalence of diabetes mellitus, impaired fasting glucose, impaired glucose tolerance, hyperlipidemia, and cardiovascular disease was significantly higher among those with hypertension (all *p* < 0.0001). Educational attainment beyond high school was less common (*p* = 0.02), and they were more likely to be married/partnered or divorced/separated/widowed (*p* < 0.0001), while income levels did not differ significantly between groups (*p* = 0.73).

**Table 1:** Basic characters of the study population.

Characteristic	Total	Non-hypertension	Hypertension	*p* value
Age, mean (SE), years	45.41 (0.38)	40.50 (0.35)	55.55 (0.42)	< 0.0001
Sex, *n* (%)				0.01
Female	3,880 (48.89)	2,440 (50.42)	1,440 (45.72)	
Male	4,174 (51.11)	2,466 (49.58)	1,708 (54.28)	
Race, *n* (%)				< 0.0001
Mexican American	1,189 (7.68)	838 (8.75)	351 (5.46)	
Non-Hispanic Black	1,486 (9.75)	766 (8.80)	720 (11.71)	
Non-Hispanic White	3,985 (70.91)	2,385 (70.31)	1,600 (72.15)	
Other	654 (6.67)	432 (6.92)	222 (6.14)	
Other Hispanic	740 (5.00)	485 (5.22)	255 (4.54)	
Marital status, *n* (%)				< 0.0001
Divorced, separated, or widowed	1,590 (15.85)	728 (12.50)	862 (22.77)	
Married or living with partner	4,968 (63.29)	3,006 (61.06)	1,962 (67.91)	
Never married	1,496 (20.86)	1,172 (26.45)	324 (9.32)	
PIR, *n* (%)				0.73
High income	2,784 (48.19)	1,711 (48.58)	1,073 (47.36)	
Middle income	3,050 (33.45)	1,828 (33.16)	1,222 (34.05)	
Low income	2,220 (18.36)	1,367 (18.26)	853 (18.58)	
Education level, *n* (%)				0.02
More than high school	4,540 (63.51)	2,867 (65.21)	1,673 (60.00)	
Completed high school	1,853 (23.91)	1,073 (22.62)	780 (26.57)	
Less than high school	1,661 (12.58)	966 (12.17)	695 (13.43)	
Drinking status, *n* (%)				< 0.0001
Heavy	1,778 (23.42)	1,259 (25.89)	519 (18.31)	
Moderate	1,372 (18.55)	919 (19.86)	453 (15.82)	
Mild	2,994 (39.74)	1,717 (37.85)	1,277 (43.63)	
Former	1,002 (9.47)	493 (7.99)	509 (12.54)	
Never	908 (8.83)	518 (8.41)	390 (9.69)	
Smoking status, *n* (%)				< 0.0001
Former	1,996 (24.13)	974 (20.70)	1,022 (31.22)	
Now	1,667 (19.17)	1,122 (20.36)	545 (16.70)	
Never	4,391 (56.70)	2,810 (58.93)	1,581 (52.08)	
PA, *n* (%)				< 0.0001
Insufficient	1,290 (14.68)	666 (12.63)	624 (18.91)	
Sufficient	6,764 (85.32)	4,240 (87.37)	2,524 (81.09)	
BMI, *n* (%), kg/m²				< 0.0001
Underweight (<18.5)	118 (1.43)	89 (1.86)	29 (0.54)	
Normal (18.5 to <25)	2,183 (29.36)	1,638 (35.06)	545 (17.59)	
Overweight (25 to <30)	2,736 (32.93)	1,688 (33.54)	1,048 (31.66)	
Obese (30 or greater)	3,017 (36.28)	1,491 (29.55)	1,526 (50.21)	
DASH score, mean (SE)	2.19 (0.02)	2.20 (0.02)	2.16 (0.04)	0.39
Energy intake, mean (SE), kcal/day	2,151.30 (15.27)	2,175.47 (19.61)	2,101.34 (19.05)	0.01
Protein intake, mean (SE), g/day	84.52 (0.69)	85.02 (0.69)	83.49 (1.07)	0.14
Carbohydrate intake, mean (SE), g/day	255.06 (1.88)	260.11 (2.73)	244.63 (2.19)	< 0.001
Sugars intake, mean (SE), g/day	112.43 (1.32)	115.44 (1.85)	106.20 (1.40)	< 0.001
Fat intake, mean (SE), g/day	83.12 (0.69)	83.48 (0.87)	82.39 (0.81)	0.30
All of flavonoids, mean (SE), mg/day	226.93 (8.32)	217.81 (9.06)	245.76 (13.03)	0.05
Isoflavones, mean (SE), mg/day	2.16 (0.17)	2.40 (0.20)	1.66 (0.26)	0.03
Anthocyanidins, mean (SE), mg/day	15.10 (0.92)	14.86 (0.94)	15.60 (1.25)	0.50
Flavan-3-ols, mean (SE), mg/day	176.96 (7.95)	168.01 (8.44)	195.46 (12.94)	0.05
Flavanones, mean (SE), mg/day	12.38 (0.43)	12.52 (0.49)	12.08 (0.73)	0.61
Flavones, mean (SE), mg/day	1.02 (0.05)	1.05 (0.07)	0.98 (0.04)	0.36
Flavonols, mean (SE), mg/day	19.30 (0.39)	18.99 (0.46)	19.96 (0.51)	0.10
DM, *n* (%)				< 0.0001
DM	1,225 (10.83)	389 (5.50)	836 (21.84)	
IFG	418 (4.66)	215 (3.74)	203 (6.54)	
IGT	249 (2.70)	125 (2.16)	124 (3.81)	
No	6,162 (81.82)	4,177 (88.60)	1,985 (67.81)	
Hyperlipidemia, *n* (%)				< 0.0001
No	2,421 (32.47)	1,817 (39.00)	604 (18.98)	
Yes	5,633 (67.53)	3,089 (61.00)	2,544 (81.02)	
CVD, *n* (%)				< 0.0001
No	7,331 (93.40)	4,745 (97.44)	2,586 (85.05)	
Yes	723 (6.60)	161 (2.56)	562 (14.95)	

### 3.2 Association between flavonoid intake and hypertension

 Table 2 presents the associations between various flavonoid compounds and hypertension risk using logistic regression models. After comprehensive adjustment for potential confounders (Model 2), total flavonoid intake demonstrated a significant inverse association with hypertension. Compared with the lowest quartile (Q1), participants in Q4 exhibited a 25% lower odds of hypertension (OR = 0.75, 95% CI: 0.60–0.93, *p* = 0.01), with a significant dose-response relationship (*p* for trend = 0.01).

**Table 2:** Association between total flavonoid intake and hypertension weighted multivariate logistic regression.

Character	Crude model		Model 1		Model 2	
	OR (95%CI)	*p*	OR (95%CI)	*p*	OR (95%CI)	*p*
All of flavonoids (mg/day)						
Q1 [0.075,27.398]	ref		ref		ref	
Q2 (27.398,70.265]	1.06 (0.82,1.39)	0.64	0.87 (0.66,1.16)	0.33	0.89 (0.63,1.26)	0.48
Q3 (70.265,237.857]	1.09 (0.87,1.37)	0.46	0.67 (0.52,0.87)	0.003	0.77 (0.58,1.03)	0.07
Q4 (237.857,6,974.47]	0.94 (0.79,1.11)	0.46	0.65 (0.54,0.79)	<0.01	0.75 (0.60,0.93)	0.01
*p* for trend		0.53		<0.01		0.01
Isoflavones (mg/day)						
Q1 [0,0]	ref		ref		ref	
Q2 (0,0.01]	0.89 (0.75,1.07)	0.21	0.81 (0.65,1.01)	0.06	0.82 (0.66,1.02)	0.07
Q3 (0.01,0.09]	0.86 (0.73,1.02)	0.08	0.79 (0.64,0.96)	0.02	0.84 (0.64,1.09)	0.17
Q4 (0.09,210.51]	0.71 (0.59,0.85)	<0.01	0.78 (0.63,0.96)	0.02	0.92 (0.71,1.19)	0.48
*p* for trend		<0.01		0.01		0.37
Anthocyanidins (mg/day)						
Q1 [0,0.141]	ref		ref		ref	
Q2 (0.141,2.315]	1.00 (0.80,1.25)	0.99	0.83 (0.64,1.09)	0.17	0.86 (0.64,1.15)	0.27
Q3 (2.315,12.669]	1.08 (0.86,1.37)	0.49	0.78 (0.59,1.02)	0.06	0.90 (0.66,1.24)	0.48
Q4 (12.669,756.1]	1.00 (0.81,1.23)	1.00	0.57 (0.46,0.72)	<0.01	0.74 (0.58,0.93)	0.01
*p* for trend		0.82		<0.01		0.02
Flavan-3-ols(mg/day)						
Q1 [0,5.581]	ref		ref		ref	
Q2 (5.581,16.807]	1.02 (0.79,1.31)	0.89	0.81 (0.61,1.08)	0.14	0.92 (0.68,1.24)	0.54
Q3 (16.807,170.515]	0.96 (0.77,1.21)	0.73	0.64 (0.50,0.82)	<0.01	0.73 (0.55,0.97)	0.03
Q4 (170.515,6,724.88]	0.91 (0.76,1.08)	0.27	0.65 (0.54,0.79)	<0.01	0.76 (0.62,0.93)	0.01
*p* for trend		0.19		<0.01		0.003
Flavanones(mg/day)						
Q1 [0,0.07]	ref		ref		ref	
Q2 (0.07,0.792]	1.01 (0.84,1.22)	0.91	0.92 (0.74,1.15)	0.44	0.96 (0.74,1.25)	0.75
Q3 (0.792,19.691]	0.97 (0.78,1.21)	0.80	0.74 (0.55,0.99)	0.04	0.89 (0.63,1.25)	0.46
Q4 (19.691,590.625]	1.01 (0.79,1.29)	0.94	0.65 (0.49,0.86)	0.003	0.74 (0.55,1.01)	0.06
*p* for trend		0.97		0.001		0.05
Flavones(mg/day)						
Q1 [0,0.21]	ref		ref		ref	
Q2 (0.21,0.555]	1.00 (0.84,1.19)	0.97	0.86 (0.69,1.07)	0.17	0.94 (0.75,1.18)	0.55
Q3 (0.555,1.14]	0.91 (0.73,1.14)	0.41	0.73 (0.58,0.93)	0.01	0.82 (0.64,1.06)	0.12
Q4 (1.14,87.245]	0.99 (0.81,1.21)	0.94	0.72 (0.57,0.90)	0.01	0.85 (0.68,1.08)	0.16
*p* for trend		0.76		0.003		0.11
Flavonols(mg/day)						
Q1 [0,7.565]	ref		ref		ref	
Q2 (7.565,13.71]	0.98 (0.79,1.23)	0.88	0.82 (0.64,1.06)	0.12	0.81 (0.61,1.09)	0.15
Q3 (13.71,23.524]	0.95 (0.79,1.14)	0.56	0.73 (0.59,0.89)	0.003	0.76 (0.60,0.98)	0.04
Q4 (23.524,262.435]	1.03 (0.86,1.24)	0.71	0.80 (0.65,0.99)	0.04	0.89(0.68,1.16)	0.34
*p* for trend		0.76		0.01		0.32

Crudel model adjusted no confounding factor; Model 1 adjusted age, sex, race; Model 2 adjusted sex, age, race, marital status, PIR, education level, drinking satus, smoking status, PA, BMI, DASH score, energy intake, protein intake, carbohydrate intake, sugars intake, fat intake, DM, Hyperlipidemia, CVD.

Among specific flavonoid subclasses, anthocyanidins and flavan-3-ols showed the most robust protective associations. For anthocyanidins, individuals in the highest intake quartile had 26% reduced odds of hypertension (OR = 0.74, 95% CI: 0.58–0.93, *p* = 0.01) compared to the lowest quartile, with a significant linear trend (*p* for trend =0.02). Similarly, flavan-3-ols demonstrated a protective gradient, with Q3 (OR = 0.73, 95% CI: 0.55–0.97, *p* = 0.03) and Q4 (OR = 0.76, 95% CI: 0.62–0.93, *p* = 0.01) both associated with significantly lower hypertension risk (*p* for trend = 0.003).

Flavanones showed a marginally significant trend (*p* for trend =0.05), with the highest intake quartile approaching statistical significance (OR = 0.74, 95% CI: 0.55–1.01, *p* = 0.06). For flavonols, only the third quartile demonstrated a significant inverse association with hypertension (OR = 0.76, 95% CI: 0.60–0.98, *p* = 0.04), but the overall trend was not significant (*p* for trend = 0.32).

While isoflavones showed significant protective associations in the crude model (*p*-trend < 0.01) and minimally adjusted model (*p* for trend =0.01), this relationship was substantially attenuated after full adjustment for confounders in model 2 (*p* for trend = 0.37). Similarly, flavones demonstrated significant associations in model 1 (*p* for trend = 0.003) but not in the fully adjusted Model 2 (*p* for trend = 0.11).

These findings suggest that higher intakes of total flavonoids—particularly anthocyanidins and flavan-3-ols—may confer protection against hypertension.

### 3.3 Overall dose-response relationship between flavonoid intake and hypertension

**Figure 2:**
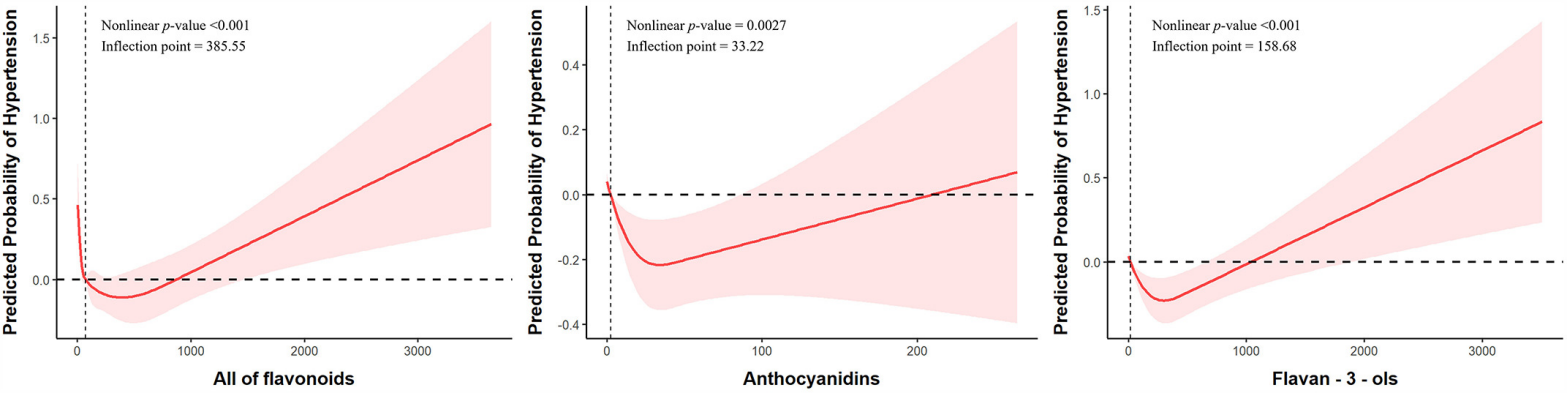
Nonlinear associations between dietary flavonoid intake and hypertension risk. Models were adjusted for sex, age, race, marital status, PIR, education level, drinking status, smoking status, PA, BMI, DASH score, energy intake, protein intake, carbohydrate intake, sugars intake, fat intake, DM, Hyperlipidemia, CVD.

As shown in Figure 2, for total flavonoid intake, a U-shaped relationship is observed with an inflection point at 385.55 mg/day. Initially, at very low intake levels, there appears to be a higher probability of hypertension which rapidly decreases and reaches its lowest point around 400–600 mg/day before gradually increasing again with higher intake levels. For anthocyanidins, an inverted U-shaped relationship is observed with an inflection point at 33.22 mg/day (95%CI: 0.60–0.98). The probability of hypertension initially decreases with increasing anthocyanidin intake, reaches its minimum at approximately 33 mg/day, and then gradually increases with higher intake levels. For flavan-3-ols, the relationship shows a J-shaped pattern with an inflection point at 158.68 mg/day. The probability of hypertension decreases with initial intake up to approximately 150–200 mg/day, after which it increases steadily with higher intake levels. These findings suggest that moderate consumption of flavonoids, particularly within the range identified by the inflection points, may be associated with lower hypertension risk, while both very low and excessive intakes may be associated with higher risk.

### 3.4 Subgroup analysis results of flavonoid and hypertension

**Figure 3:**
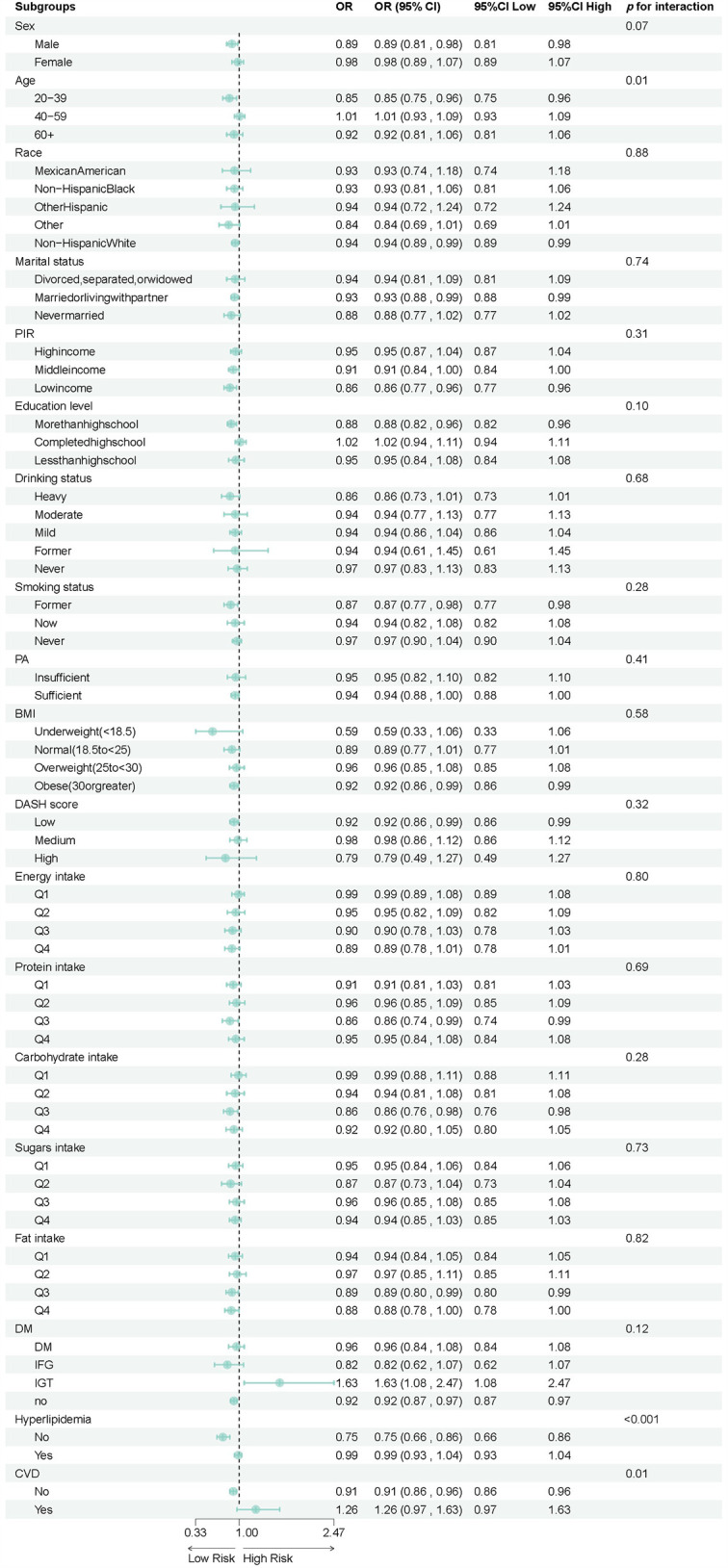
Forest plot of stratified analysis of the association between hypertension and total flavonoid.

Subgroup analyses revealed heterogeneity in the association between total flavonoid intake and hypertension risk across different populations, as shown in Table 3 and Figure 3. Age-stratified analysis demonstrated a significant interaction effect (*p* for interaction = 0.01), with younger adults aged 20–39 years (OR = 0.85, 95%CI: 0.75–0.96) showing a clear protective effect from total flavonoid intake. In contrast, adults aged 40–59 years (OR = 1.01, 95%CI: 0.93–1.09) exhibited no protective effect, while those aged 60 and above (OR = 0.92, 95%CI: 0.81–1.06) showed a non-statistically significant protective trend. Metabolic health status significantly modified the association with total flavonoid intake. Hyperlipidemia status demonstrated a highly significant interaction effect (*p* for interaction <0.0001), where individuals without hyperlipidemia (OR = 0.75, 95%CI: 0.66–0.86) experienced a 25% reduction in hypertension risk from flavonoid intake, while those with hyperlipidemia (OR = 0.99, 95%CI: 0.93–1.04) received virtually no protective benefit. Similarly, cardiovascular disease status showed a significant interaction (*p* for interaction = 0.01), with those free of cardiovascular disease (OR = 0.91, 95%CI: 0.86–0.96) demonstrating protective effects, whereas patients with cardiovascular disease (OR = 1.26, 95%CI: 0.97–1.63) showed a trend toward increased risk, though not reaching statistical significance. No significant interaction effects were observed across subgroups of sex, race, marital status, income level, education level, alcohol consumption, smoking status, physical activity level, BMI, DASH score, or energy and nutrient intake levels (all *p* for interaction >0.05).

**Table 3:** Subgroup analysis between hypertension and total flavonoid.

Subgroups	OR (95%CI)	*p* for interaction
Sex		0.07
Male	0.89 (0.81,0.98)	
Female	0.98 (0.89,1.07)	
Age		0.01
20–39	0.85 (0.75,0.96)	
40–59	1.01 (0.93,1.09)	
60+	0.92 (0.81,1.06)	
Race		0.88
Mexican American	0.93 (0.74,1.18)	
Non-Hispanic Black	0.93 (0.81,1.06)	
Other Hispanic	0.94 (0.72,1.24)	
Other	0.84 (0.69,1.01)	
Non-Hispanic White	0.94 (0.89,0.99)	
Marital status		0.74
Divorced, separated, or widowed	0.94 (0.81,1.09)	
Married or living with partner	0.93 (0.88,0.99)	
Never married	0.88 (0.77,1.02)	
PIR		0.31
High income	0.95 (0.87,1.04)	
Middle income	0.91 (0.84,1.00)	
Low income	0.86 (0.77,0.96)	
Education level		0.10
More than high school	0.88 (0.82,0.96)	
Completed high school	1.02 (0.94,1.11)	
Less than high school	0.95 (0.84,1.08)	
Drinking status		0.68
Heavy	0.86 (0.73,1.01)	
Moderate	0.94 (0.77,1.13)	
Mild	0.94 (0.86,1.04)	
Former	0.94 (0.61,1.45)	
Never	0.97 (0.83,1.13)	
Smoking status		0.28
Former	0.87 (0.77,0.98)	
Now	0.94 (0.82,1.08)	
Never	0.97 (0.90,1.04)	
PA		0.41
Insufficient	0.95 (0.82,1.10)	
Sufficient	0.94 (0.88,1.00)	
BMI		0.58
Underweight (<18.5)	0.59 (0.33,1.06)	
Normal (18.5 to <25)	0.89 (0.77,1.01)	
Overweight (25 to <30)	0.96 (0.85,1.08)	
Obese (30 or greater)	0.92 (0.86,0.99)	
DASH score		0.32
Low	0.92 (0.86,0.99)	
Medium	0.98 (0.86,1.12)	
High	0.79 (0.49,1.27)	
Energyintake		0.80
Q1	0.99 (0.89,1.08)	
Q2	0.95 (0.82,1.09)	
Q3	0.90 (0.78,1.03)	
Q4	0.89 (0.78,1.01)	
Proteinintake		0.69
Q1	0.91 (0.81,1.03)	
Q2	0.96 (0.85,1.09)	
Q3	0.86 (0.74,0.99)	
Q4	0.95 (0.84,1.08)	
Carbohydrateintake		0.28
Q1	0.99 (0.88,1.11)	
Q2	0.94 (0.81,1.08)	
Q3	0.86 (0.76,0.98)	
Q4	0.92 (0.80,1.05)	
Sugarsintake		0.73
Q1	0.95 (0.84,1.06)	
Q2	0.87 (0.73,1.04)	
Q3	0.96 (0.85,1.08)	
Q4	0.94 (0.85,1.03)	
Fatintake		0.82
Q1	0.94 (0.84,1.05)	
Q2	0.97 (0.85,1.11)	
Q3	0.89 (0.80,0.99)	
Q4	0.88 (0.78,1.00)	
DM		0.12
DM	0.96 (0.84,1.08)	
IFG	0.82 (0.62,1.07)	
IGT	1.63 (1.08,2.47)	
No	0.92 (0.87,0.97)	
Hyperlipidemia		<0.0001
No	0.75 (0.66,0.86)	
Yes	0.99 (0.93,1.04)	
CVD		0.01
No	0.91(0.86,0.96)	
Yes	1.26(0.97,1.63)	

The significant association observed in the IGT subgroup (OR:1.63) should be interpreted with caution, as it may be influenced by residual confounding or the relatively small sample size. This raises the possibility of a type I error, and the result should be considered exploratory.

## 4 Discussion

This cross-sectional analysis, employing data from 8,054 participants from the NHANES database spanning 2007–2010 and 2017–2018, explored the association between total flavonoids and its six subclasses with hypertension risk. After multivariate adjustment, results demonstrated a significant inverse correlation between total flavonoid intake and hypertension, with the highest intake quartile (Q4) showing a 25% reduced risk compared to the lowest quartile (Q1) (OR = 0.75, 95% CI: 0.60–0.93, *p* = 0.01). Particularly, anthocyanidins and flavan-3-ols exhibited the strongest protective effects, with the highest intake groups reducing hypertension risk by 26% (OR = 0.74, 95% CI: 0.58–0.93, *p* = 0.01) and 24% (OR = 0.76, 95% CI: 0.62–0.93, *p* = 0.01), respectively. Dose-response analysis further revealed nonlinear relationships between flavonoid intake and hypertension, suggesting that moderate consumption of flavonoid compounds may be more beneficial for cardiovascular health.

Our findings align with several existing studies. A comprehensive systematic review and meta-analysis by Lagou et al. examining 145 randomized controlled trials demonstrated that flavan-3-ol-rich interventions significantly reduced office blood pressure (−2.8/-2.0 mmHg) and 24 h ambulatory blood pressure (−3.7/-2.6 mmHg) with more pronounced effects in hypertensive individuals (−5.9/-2.7 mmHg), strongly corroborating our results (16). Similarly, Igwe et al. showed that anthocyanin-rich Queen Garnet plum juice significantly reduced systolic BP, diastolic BP, mean arterial pressure, and heart rate (*p* < 0.05) in participants regardless of dosing regimen while having no effect on cognitive function, further supporting our conclusions about anthocyanidins’ protective effects (17). Cassidy et al. found that participants in the highest quintile of anthocyanin intake had an 8% reduction in hypertension risk compared to those in the lowest quintile, with a more pronounced 12% risk reduction in adults ≤60 years (18). Their findings suggest anthocyanins may prevent hypertension through vasodilatory properties related to specific structural characteristics. This aligns with our stratified analysis, where the 20–39 age group demonstrated the most significant protective effect from flavonoid intake.

The subgroup analysis yielded significant insights into the relationship between flavonoid intake and hypertension. Age notably modified this association (*p* for interaction = 0.01), aligning with previous findings by Cassidy et al. (18). Importantly, our study revealed that metabolic health status substantially modifies flavonoid's protective effects, with hyperlipidemia status showing a particularly strong interaction (*p* < 0.0001). In individuals without hyperlipidemia, flavonoid intake reduced hypertension risk by 25% (OR = 0.75, 95%CI: 0.66–0.86), while those with hyperlipidemia received negligible protection (OR = 0.99, 95%CI: 0.93–1.04). This differential response supports earlier research demonstrating that polyphenolic compounds exert stronger antioxidant and anti-inflammatory effects in populations without metabolic disturbances (19–21).

The dose-response analysis further uncovered nonlinear relationships between flavonoid intake and hypertension, consistent with findings by Kong et al. and Lin et al. (22, 23). These nonlinear relationships suggest the “moderation is optimal” principle, where inadequate or excessive intake fails to provide optimal protection. Rees et al. demonstrated that flavonoid-rich blueberries affect endothelial function in a dose-dependent manner that plateaus at 766 mg total polyphenols. Exceeding this threshold may counteract the beneficial effects, indicating an optimal therapeutic window rather than a simple “more is better” relationship (24). These findings have significant implications for individualized dietary guidance and hypertension prevention strategies, emphasizing the importance of adjusting flavonoid intake based on age and metabolic status.

The molecular mechanisms underlying the association between flavonoid compounds and hypertension are complex and multifaceted, involving multiple physiological and pathological pathways. As secondary metabolites widely present in plants, flavonoids exhibit structural diversity and demonstrate a rich array of biological activities, including antioxidative, anti-inflammatory, and antithrombotic properties (25–27). In the context of hypertension, these bioactivities can directly influence vascular function and blood pressure regulation.

Central to flavonoids’ hypotensive effects is their enhancement of vascular endothelial function. By stimulating nitric oxide production—a potent vasodilator—flavonoids effectively reduce vascular tension and regulate blood pressure (28–30). This endothelial-dependent mechanism is complemented by flavonoids’ remarkable antioxidant properties, which shield vascular tissues from oxidative damage and preserve arterial elasticity (31–33). These dual actions likely underpin our observation.

Flavonoids further modulate blood pressure through attenuation of sympathetic nervous system activity, thereby reducing cardiac output and peripheral vascular resistance (34). This neurogenic regulation works synergistically with their anti-inflammatory properties, which mitigate the vascular inflammation implicated in hypertension pathogenesis (35–37). Additionally, certain flavonoids exhibit angiotensin-converting enzyme inhibitory effects similar to conventional antihypertensive medications, directly dampening the renin-angiotensin-aldosterone system that governs blood pressure homeostasis (38–40).

The main strengths of our study include the use of the nationally representative NHANES database with a large sample size (8,054 participants), providing substantial statistical power. We employed rigorous statistical analysis strategies with multiple progressively adjusted models that effectively controlled for potential confounding factors. Through restricted cubic spline regression analysis, we revealed nonlinear relationships between flavonoid intake and hypertension that extend beyond simple linear assumptions. Furthermore, we analyzed not only total flavonoid intake but also the specific effects of six major flavonoid subclasses, and systematically explored the modifying effects of metabolic health status on the flavonoid-hypertension relationship, providing valuable references for dietary guidance and hypertension prevention strategies.

Despite these strengths, our study has several important limitations. The cross-sectional design only allows for detecting associations without establishing causality. Although NHANES data is nationally representative, participants were limited to American adults, restricting the extrapolation of results to other racial and regional populations. Flavonoid intake assessment based on two-day 24-hour dietary recall questionnaires may introduce recall bias and might not fully reflect long-term dietary habits. Additionally, despite adjusting for multiple known confounding factors, unmeasured confounders may still exist, such as genetic polymorphisms and gut microbiota composition, which could influence flavonoid metabolism and hypertension risk. These limitations suggest caution when interpreting and applying our findings.

## 5 Conclusion

Our study reveals a significant inverse relationship between dietary flavonoid intake and hypertension risk, with anthocyanidins and flavan-3-ols showing the strongest protective effects. These associations follow nonlinear patterns, suggesting moderate consumption may be more beneficial than high doses. These insights enhance our understanding of cardiovascular protection from plant-based foods and inform more precise dietary guidelines. The observed modifications by age and metabolic status highlight individual differences in flavonoid metabolism, opening avenues for precision nutrition. Despite the cross-sectional design limiting causal inference, our findings, together with existing mechanistic studies, offer valuable strategies for hypertension prevention. As global hypertension rates rise, incorporating flavonoid-rich foods represents a simple yet effective preventive approach. Future research should validate these associations prospectively, explore synergistic effects between flavonoid subclasses, and investigate flavonoid-medication interactions to optimize cardiovascular health management.

## Data Availability

The datasets presented in this study can be found in online repositories. The names of the repository/repositories and accession number(s) can be found in the article/Supplementary Material.
